# Targeted Therapy Followed by Salvage Surgery and Adjuvant Therapy: A Promising Therapy for Lung Cancer With Malignant Pleural Effusion From a Case Report

**DOI:** 10.3389/fsurg.2021.659983

**Published:** 2021-12-10

**Authors:** Han-Yu Deng, Deyan Li, Ying Ren, Ke Wang, Xiaojun Tang

**Affiliations:** ^1^Lung Cancer Center, West China Hospital, Sichuan University, Chengdu, China; ^2^Operating Room, West China Hospital, Sichuan University, Chengdu, China; ^3^Department of Outpatient, West China Hospital, Sichuan University, Chengdu, China; ^4^Department of Respiratory and Critical Care Medicine, West China Hospital, Sichuan University, Chengdu, China

**Keywords:** lung cancer, malignant pleural effusion, targeted therapy, salvage surgery, adjuvant therapy

## Abstract

**Introduction:** Malignant pleural effusion was encountered in about 8–15% of lung cancer patients at initial cancer diagnosis. The optimal therapeutic strategies for lung cancer with malignant pleural effusion (MPE) remain unclear.

**Case Description:** In this study, we reported a case of lung cancer with MPE, which was successfully managed with a multidisciplinary therapeutic strategy. The patient initially received gefitinib for 4 months with excellent response and he underwent salvage thoracoscopic lobectomy and systematic lymphadenectomy. Pathological complete response was confirmed for the patient and he discontinued gefitinib but received 4 cycles of adjuvant chemotherapy instead. The patient is still alive without disease progression for 62 months after surgery.

**Conclusions:** Combining targeted therapy, salvage surgery, and adjuvant therapy may be a promising treatment strategy for lung cancer with MPE harboring oncogene-targeted mutations.

## Introduction

Malignant pleural effusion is commonly encountered in about 8–15% of lung cancer patients at the time of initial cancer diagnosis, which has been categorized as stage IVa disease in the eighth edition of the tumor-node-metastasis staging system (1). The prognosis of lung cancer patients with malignant pleural effusion (MPE) remains dismal with a median overall survival time of 5 months and a 5-year survival rate of 3% (2). Current guidelines recommended non-surgical therapy including local therapy (for example ambulatory small catheter drainage, pleurodesis, and pericardial window) with similar treatment strategies to other stage IV diseases consisting of systemic therapy and palliative therapy (3). In this study, we reported a case of lung cancer with MPE, which was successfully managed with the combined therapeutic strategy of targeted therapy followed by salvage surgery and adjuvant therapy.

## Case Report

**Figure 1:**
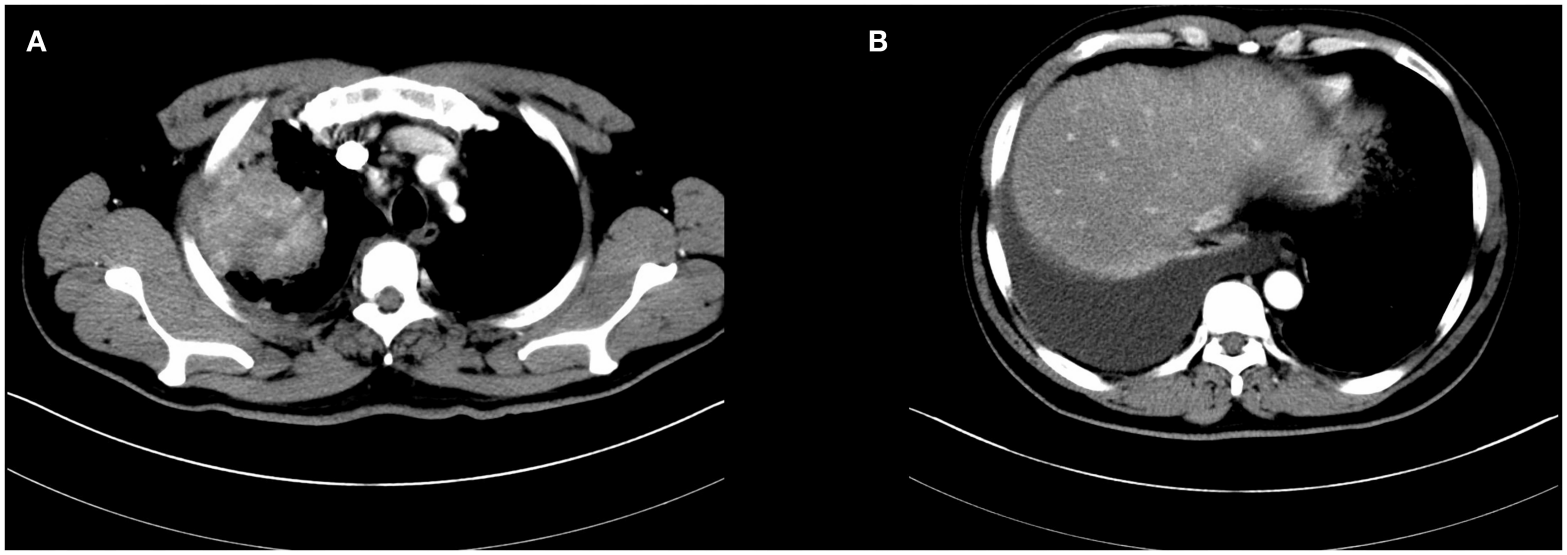
Initial chest computed tomography of the patient revealed a large mass in the right upper lobe with enlarged mediastinal lymph node and malignant pleural effusion **(A,B)**.

**Figure 2:**
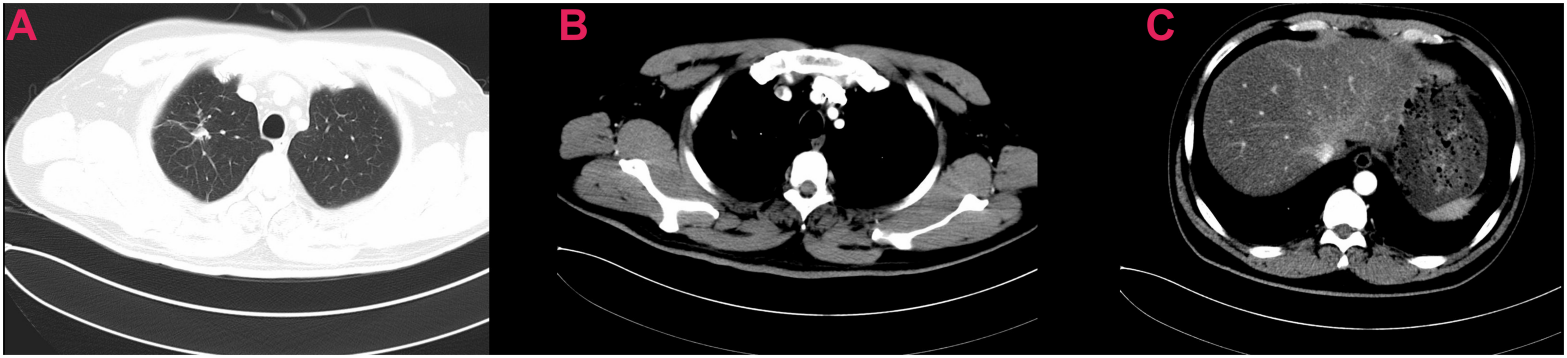
Preoperative chest computed tomography of the patient showing excellent response to neoadjuvant targeted therapy **(A–C)**.

**Figure 3:**
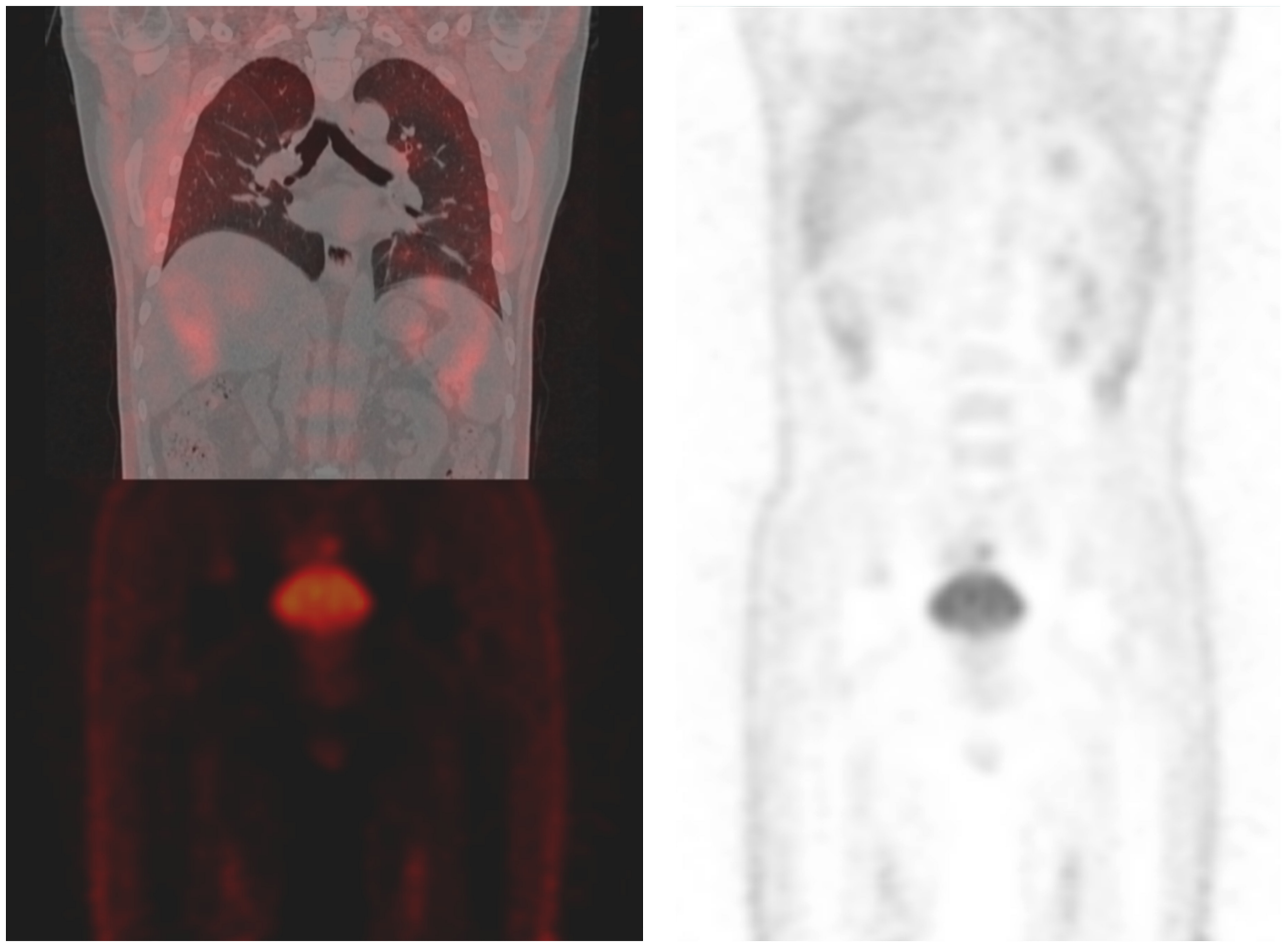
Recent positron emission tomography/computed tomography of the patient shows no sign of recurrence.

**Figure 4:**
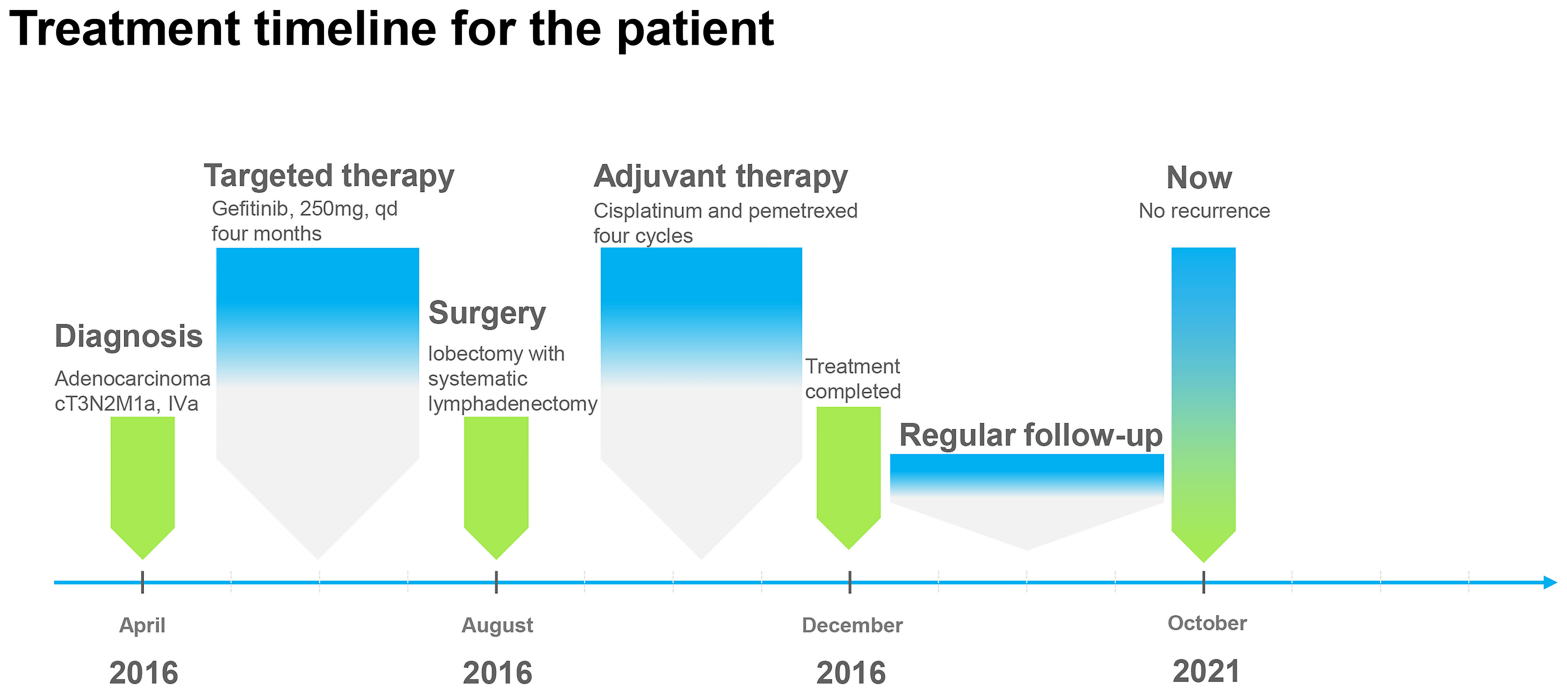
Treatment timeline of the patient in our case.

In April 2016, a 51-year old male patient complained of consistent cough for 2 months and was admitted to our center for a diagnosis of poorly differentiated lung adenocarcinoma [PCK(+), CK7 (focally +), TTF-1(+), CK18(+), CK5/6(-), P63(scattered +), CK14(-), CDX-2(-), CD56(+), CgA(-), Sgn(-), Ki-67(~50%)] in the right upper lobe with enlarged ipsilateral mediastinal lymph node and MPE confirmed by cytological examination of the fluid *via* both cell block and smear from the collected sample by thoracentesis, which was not further confirmed by pleural biopsy (cT3N2M1a, IVa) (Figure 1). The patient was generally in normal condition but was found to have type two diabetes mellitus with an Eastern Cooperative Oncology Group score of 1. With the primary tumor extracted *via* percutaneous needle biopsy for next-generation sequencing, it was confirmed to have epidermal growth factor receptor (EGFR) gene mutation (exon 21 L858R) and the patient was advised to receive gefitinib (250 mg, QD) for treatment. After taking gefitinib for 4 months, the patient was re-evaluated comprehensively and an excellent radiographic response to gefitinib was found on his chest computed tomography scan (Figure 2). Therefore, the patient was discussed in a multidisciplinary meeting in our center, and salvage surgery was recommended for him. In August 2016, after providing signed informed consent, the patient received lobectomy and systematic lymph node dissection under video-assisted thoracoscopic surgery (VATS) successfully and intraoperative findings did not reveal any pleural involvement, which was further confirmed by pleural biopsy. The patient was discharged on postoperative day 5 uneventfully and his postoperative pathological finding revealed no residual tumor neither in the right upper lobe nor in the mediastinal lymph node and pathological complete response to gefitinib was confirmed in the patient (ypT0N0M0) as shown in his pathological report that numerous chronic inflammatory cells, foamy histiocytes, and dense fibrosis were observed and no viable tumor was seen [PCK(-), EMA(-), CK7(-), TTF-1(-), NapsinA(-), CK5/6(-), P63(-), PGM-1 (inflammatory cells+), complete pathologic response]. As usual, after surgery, the patient discontinued gefitinib and underwent four cycles of adjuvant chemotherapy (cisplatinum 40 mg on day 1 to 3 and pemetrexed 800 mg on day 1) instead because chemotherapy was the regular regimen for postoperative adjuvant therapy then and finished his last course of adjuvant chemotherapy in December 2016 with only grade 2 of leukopenia without any drug-related grade 3 to 4 adverse events. Since then, the patient received no additional treatment (such as targeted therapy, chemotherapy, or radiotherapy) and was followed up regularly every 3–4 months with chest and abdominal CT scans and tumor biomarkers and annual positron emission tomography (PET)/CT scan. The patient's recent PET/CT scan (October 2021) still revealed no sign of recurrence (Figure 3), and he is still alive without disease for 62 months after surgery (disease-free survival: 62 months). The whole treatment timeline of the patient in our case was summarized in Figure 4.

## Discussion

Previous studies have shown that surgery could benefit certain carefully selected patients with pleural metastasis and the reported 5-year OS of these patients treated with surgical resection ranged from 16 to 31% (4). Our previous study also indicated that surgical resection of the primary tumor could bring survival benefits for patients with unexpected pleural metastasis found during operation (5). However, it should be noted that patients with MPE yielded significantly worse survival than those without after surgery for pleural metastasis (6), suggesting that MPE may represent diffused pleural dissemination and surgery may be precluded for MPE (7). However, previous studies applied surgery with systemic therapy for treating advanced lung cancer with MPE and found that the long-term outcomes of combined therapy for these patients remain conflicting as some found that surgery with systemic therapy could benefit patients while others found a worse outcome after surgery (8). Therefore, for MPE, induction therapy followed by salvage surgery and subsequent systemic therapy was investigated. However, most of the previous studies applied chemoradiotherapy for induction therapy in patients with MPE and the rate of complete response was extremely low (9, 10). Moreover, the majority of these patients after surgery relapsed during follow-up (9). Therefore, the role of salvage surgery in treating advanced lung cancer with MPE remains further to be elucidated.

Similar to the dilemma encountered in neoadjuvant therapy for stage III lung cancer (11), the optimal induction regimens for lung cancer with MPE remain far from being established. As the promising effects of oncogene-targeted therapy for lung cancer, targeted therapy has already become the first-line therapy for advanced lung cancer harboring sensitizing mutations. Considering that EGFR tyrosine kinase inhibitors (TKIs) could yield a significantly higher response rate than chemoradiotherapy and confer survival benefit over chemoradiotherapy in advanced lung cancer harboring sensitizing mutations (12), in our case, the patient received gefitinib as the initial treatment because of the EGFR gene mutation. Surprisingly, the patient showed complete response to gefitinib after 4-month treatment. As we all know, the majority of patients receiving the first generation of EGFR-TKIs will progress within 1 year due to resistance mutation (12). Therefore, in our case, salvage surgery was recommended because of the radiographic finding of complete response. Moreover, we have successfully performed VATS lobectomy with systematic lymphadenectomy for the patient. Because of no residual tumor in the right upper lobe and mediastinal lymph node (ypT0N0), the patient only received 4 cycles of adjuvant chemotherapy without postoperative targeted therapy (as discussed by the multidisciplinary team considering that no residual tumor was revealed in the patient) for minimizing postoperative recurrence and was regularly followed up thereafter. And the patient is still alive without any sign of recurrence or metastasis for nearly 62 months. Therefore, this is an interesting case in a stage IVa lung cancer patient, who was managed successfully with a therapeutic combination of targeted therapy, salvage surgery, and adjuvant therapy.

Previously, Kubo et al. (13) reported a total of 7 cases of lung cancer with MPE treated with gefitinib, who responded effectively to gefitinib and chest drainage. However, the time to treatment failure for these patients was about 0.2–19.0 months. Tsai et al. (14) reported a case with stage IV oligometastatic adenocarcinoma of the lung successfully managed with neoadjuvant afatinib (pathological complete response) followed by surgery and adjuvant afatinib as well as radiotherapy for oligometastasis (the third lumbar vertebra) and the patient was alive for 32 months after initial diagnosis. A previous study also confirmed that salvage surgery after targeted therapy could serve as a promising therapeutic option for advanced lung cancer (15). Therefore, targeted therapy may serve as induction therapy for lung cancer with MPE. Arrieta et al. (10) reported two cases of lung cancer patients with malignant pleural effusion but without extra-thoracic disease, who were treated with neoadjuvant targeted therapy followed by salvage surgery and adjuvant targeted therapy. One case was treated with erlotinib and was alive without disease for 32 months while another was treated with afatinib and was alive without disease for 28.25 months. Song et al. (15) reported three cases of lung cancer with malignant pleural effusion treated with targeted therapy followed by salvage surgery and found that the postoperative survival was 6–44 months, proving that salvage surgery after targeted therapy was feasible and promising for treating advanced lung cancer with malignant pleural effusion. Li et al. (16) also reported a case of advanced lung cancer with malignant pleural effusion treated with salvage surgery followed by targeted therapy who finally developed progression of pubic bone metastasis after 13 months after surgery. Here we summarized these similar cases of lung cancer with malignant pleural effusion successfully managed with targeted therapy followed by salvage surgery in Table 1. Therefore, taking our case together, we believe that the combination of targeted therapy followed by salvage surgery and adjuvant therapy seems to be a promising therapeutic strategy for lung cancer with MPE harboring oncogene-targeted mutations.

**Table 1:** Literature review for case reports regarding targeted therapy followed by salvage surgery for treating lung cancer with malignant pleural effusion.

**Case**	**Gender**	**Age**	**Histology**	**Clinical stage**	**Driver gene**	**Targeted therapy**	**Drug duration**	**Therapeutic response**	**Postoperative stage**	**Postoperative treatment**	**Status**	**PFS (months)**	**OS (months)**	**References**
1	Female	65	AD	cTxN2M1a	EGFR, 19del	Erlotinib	5 cycles	SD	NA	Erlotinib	Alive without progression	32	32	(10)
2	Female	63	AD	cTxN2M1a	EGFR, 19del	Afatinib	6 cycles	SD	NA	Afatinib	Alive without progression	28.25	28.25	(10)
3	Female	63	AD	cT2N0M1a	EGFR, 19del	Icotiinib	46 months	PR	pT2bN0M0	Icotinib	NA	NA	44	(15)
4	Male	45	AD	cT2N0M1a	EGFR, 19del	Gefitinib	6 months	PR	pT2aN2M0	Osimertinib	NA	NA	27	(15)
5	Female	56	AD	cT2N2M1a	ALK translocation	Crizotinib	8 months	PR	pT1aN2M0	Crizotinibi	NA	NA	6	(15)
6	Female	59	AD	cT1cN0M1a	EGFR, 19del	Gefitinib	7 months	PR	pT1bN0M0	Gefitinib	Progression of pubic bone metastasis	13	NA	(16)
7	Male	51	AD	cT3N2M1a	EGFR, L858R	Gefitinib	4 months	PR	pT0N0M0	Chemotherapy	Alive without progression	62	62	Our case

*PFS, progression-free survival; OS, overall survival; AD, adenocarcinoma; EGFR, epidermal growth factor receptor; SD, stable disease; NA, not available; PR, partial response; ALK, anaplastic lymphoma kinase*.

However, several limitations existed in our case report. First, we drew our conclusions based on only one case, which could decrease the evidence level of our conclusions. Second, our case was diagnosed with MPE only confirmed by cytological examination of the fluid without thoracoscopic pleural biopsy, which may lead to false-positive results. Therefore, for such patients, a well-detailed algorithm should be designed to ensure proper patient selection in the future. Moreover, expanding surgical indications for patients with MPE may carry significant risks, such as perioperative morbidity and mortality as well as postoperative recurrence and metastasis, and should be done only after great deliberation and thoughtful consideration of all risks. In our opinion, the salvage surgery may be considered for patients with MPE, whose tumors showed a significant radiographic response (CT or PET/CT) to initial therapy with the disappearance of pleural effusion. However, the widely accepted criteria to decide salvage surgery for patients with MPE remains further to be established. Therefore, our conclusions should be taken with caution and further similar cases are encouraged to add evidence to our conclusions.

## Conclusion

Lung cancer with MPE has an extremely poor prognosis and targeted therapy followed by salvage surgery and adjuvant therapy seems to be a promising therapeutic strategy for lung cancer with MPE harboring oncogene-targeted mutations.

## Data Availability Statement

The original contributions presented in the study are included in the article/supplementary material, further inquiries can be directed to the corresponding author/s.

## Ethics Statement

The studies involving human participants were reviewed and approved by West China Hospital, Sichuan University. The patients/participants provided their written informed consent to participate in this study.

## Author Contributions

H-YD and YR collected data and drafted the manuscript. XT, DL, and KW designed the study and revised the manuscript. All authors read and approved the final manuscript.

## Conflict of Interest

The authors declare that the research was conducted in the absence of any commercial or financial relationships that could be construed as a potential conflict of interest.

## Publisher's Note

All claims expressed in this article are solely those of the authors and do not necessarily represent those of their affiliated organizations, or those of the publisher, the editors and the reviewers. Any product that may be evaluated in this article, or claim that may be made by its manufacturer, is not guaranteed or endorsed by the publisher.
